# Optimization of the Spray-Drying Encapsulation of Sea Buckthorn Berry Oil

**DOI:** 10.3390/foods12132448

**Published:** 2023-06-22

**Authors:** Patricija Čulina, Zoran Zorić, Ivona Elez Garofulić, Maja Repajić, Verica Dragović-Uzelac, Sandra Pedisić

**Affiliations:** 1 Faculty of Food Technology and Biotechnology, University of Zagreb, P. Kasandrića 3, 23000 Zadar, Croatia; 2 Faculty of Food Technology and Biotechnology, University of Zagreb, Pierottijeva 6, 10000 Zagreb, Croatia

**Keywords:** spray drying, sea buckthorn (*Elaeagnus rhamnoides* (L.) A. Nelson) berry oil, drying temperature, carrier-to-oil ratio, gum arabic, β-cyclodextrin

## Abstract

The aim of this study was to evaluate the effect of spray-drying parameters on the physicochemical properties of encapsulated sea buckthorn berry oil. Different carriers (gum arabic, β-cyclodextrin, and their mixture (1:1, *w*/*w*)), inlet air temperatures (120, 150, and 180 °C), and carrier-to-oil ratios (2, 3, and 4, *w*/*w*) were evaluated. The obtained powders were characterized in terms of the product yield (36.79–64.60%), encapsulation efficiency (73.08–93.18%), moisture content (0.23–3.70%), hygroscopicity (1.5–7.06 g/100 g), solubility (19.55–74.70%), bulk density (0.25–0.44 g/L), total carotenoid content (mg/100 g dm), and antioxidant capacity (871.83–1454.39 μmol TE/100 g dm). All physicochemical properties were significantly affected by the carrier-to-oil ratio and inlet air temperature. Higher carrier-to-oil ratios increased the product yield, encapsulation efficiency, solubility, and bulk density and decreased the powder hygroscopicity. Elevating the drying temperatures during spray drying also increased the product yield, encapsulation efficiency, and solubility, while it decreased the powder moisture content, total carotenoid content, and antioxidant capacity. Based on the physicochemical properties, the use of β-cyclodextrin as a carrier, a drying temperature of 120 °C, and a carrier-to-oil ratio of 4 were selected as optimal conditions for the production of sea buckthorn berry oil powder. The obtained powder is a valuable material for a wide range of applications in the food and nutraceutical industries.

## 1. Introduction

Sea buckthorn (*Elaeagnus rhamnoides* (L.) A. Nelson) berry oil (SBO) is known as a rich source of lipophilic bioactive molecules (BAMs), such as polyunsaturated fatty acids, tocopherols, carotenoids, and sterols [1], with beneficial biological effects, and its use in various food formulations is increasingly being investigated. Sea buckthorn has a high nutritional value and a unique flavor due to its content of volatile compounds, and it is widely used in the baking, dairy, beverage, and confectionery industries [2]. However, the use of sea buckthorn oil in the food industry is limited due to its low solubility, sensitivity to light and heat, susceptibility to oxidative deterioration, and consequent production of an unpleasant taste [3]. The degradation of vegetable oils could be largely overcome with various encapsulation methods that protect different food components or BAMs of the oils from adverse environmental conditions or interactions with the food matrix and control their release during storage [3]. In addition, it is important to produce powders with desirable physical properties (moisture content, hygroscopicity, bulk density, solubility, etc.), as this affects their storage, packaging, and behavior in food formulations. Among the encapsulation methods, spray drying (SD) of vegetable and essential oils, fats, flavors, and colorants has attracted attention due to its low operating cost, easy production scale-up, high production yield, and resultant powders with good reconstitution characteristics, low water activity, and storage suitability [4]. SD retains the oil in the protective layer of the carrier or its mixtures, which increases the stability of bioactive oil molecules and allows easier incorporation into functional products, especially for hydrophobic compounds. The basic principle of oil SD is to dissolve the oil and carrier to produce a stable oil-in-water emulsion and then evaporate the water during drying [3]. Surfactants—also called emulsifiers—are often required to produce fine and stable emulsions during SD. A widely used nonionic surfactant that is suitable for the preparation of oil-in-water emulsions is Tween 20 [5].

Many factors can affect the encapsulation efficiency during SD, such as the type of carrier, the carrier-to-oil ratio, the properties of the emulsion obtained, and the drying conditions used [6]. High encapsulation efficiency and desirable physicochemical properties of the dried powders, such as bulk density, solubility, hygroscopicity, moisture content, retention of BAMs, and antioxidant capacity, depend significantly on the choice of carrier material and SD temperatures [7,8].

The most commonly used carrier material for the encapsulating oils is gum arabic (GA), a heteropolysaccharide composed of high branches linked by 1,6-galactopyranose residues containing galactose, arabinofuranose, arabinopyranose, rhamnopyranose, glucuronic acid, and 4-*O*-methylglucuronic acid [9]. This carrier has excellent emulsifying and film-forming properties [10], exhibits high solubility and low viscosity in aqueous solutions, and has satisfactory retention properties that facilitate the spray-drying process [9,10]. GA has an amphiphilic character, as it contains about 2% polypeptides, the hydrophobic polypeptide chains are linked to the hydrophilic polysaccharide fragments, and the hydrophobic part adsorbs strongly on the surface of the oil droplets, while the hydrophilic branches extend into the solution and limit droplet aggregation and coalescence via steric and/or electrostatic repulsion [11]. GA is ideal for encapsulating lipid droplets because it serves the function of both a surfactant and a dry matrix, and it prevents the loss of volatiles to the atmosphere. However, fluctuations in the supply of GA and its increasing price are leading to demand for alternative carrier materials that can be used instead of or in combination with GA and that can reduce the cost of encapsulating oils and flavors. In addition, mixtures of GA with other carriers can provide an encapsulation matrix with improved emulsion stability, flavor retention, and oxidation protection properties [9,12]. Among other carbohydrate-based carriers, β-cyclodextrin (β-CD) is widely used in encapsulation studies for various food applications, especially to improve the water solubility, permeability, and stability of lipophilic compounds and to enhance sensory quality [13]. It has been reported that β-CD is more thermostable and better under humid conditions than starch, and when compared to other carriers, it enables the preservation of flavor quality to a greater extent and for a longer period of time, thus ensuring the longevity of the food product [13]. The β-CD consists of seven glucopyranose units in the form of a truncated cone. The main property of cyclodextrins is that their specific steric arrangement of the hydrophilic outer surface improves the solubility of cyclodextrins in water, and the hydrophobic inner cavity forms inclusion complexes with a variety of lipophilic molecules and produces stable emulsions [13,14]. Many studies have shown that higher carrier concentrations favor the formation of powders with higher encapsulation efficiency, product yield, and bulk density, as well as lower hygroscopicity and moisture content, and they enable better retention of BAMs and antioxidant capacity [4,15].

The inlet air temperature—known as the drying temperature—is also an important parameter contributing to the efficiency of the encapsulation process. It usually ranges from 140 to 240 °C, and it affects not only the BAM content, but also the physical properties of powders, such as their water content, hygroscopicity, particle size, etc. [16]. Generally, a higher drying temperature favors the formation of powders with desirable physical properties and a higher product yield [17,18,19]. However, if the temperature is too high, it can cause heat damage to the product, with excessive bubble growth and surface defects, which increase the SD losses of BAMs [6], while too low of a drying temperature will not allow the product to dry sufficiently, leading to agglomeration of the powder in the drying chamber and dripping of the feed emulsion [19,20].

Thus, optimization of the SD conditions is recommended for the production of stable and high-quality powders with good physicochemical properties, rehydration capacity, and characteristic sensory attributes [18]. For stability and storage of powder, properties such as moisture content, water activity, and hygroscopicity are crucial, while bulk density is important for packaging [15]. Although many studies on the encapsulation of oils via SD can be found in the literature, there is limited comprehensive information on the effects of drying conditions on encapsulation of SBO by SD. Therefore, the aim of this study was to optimize the SD process of SBO (*Elaeagnus rhamnoides* (L.) A. Nelson) in terms of the carrier material selection (GA, β-CD, and their mixture), carrier-to-oil ratio (2–4), and drying temperature (120–180 °C), as well as to determine their influence on the physicochemical properties of the obtained sea buckthorn oil powders.

## 2. Materials and Methods

### 2.1. Materials

SBO obtained via supercritical CO_2_ extraction was used in this study. Supercritical CO_2_ extraction of oil from sea buckthorn (SB) berries was performed in an SFE system at an extraction pressure of 276 bar, a temperature of 35 °C, and a mass flow rate of 2 kg/h according to the method of Pavlović et al. (2016) [21] with minor modifications. The extraction process lasted 90 min until the total amount of oil was extracted (every 15 min, the amounts of extracts obtained were collected in previously weighed glass tubes and weighed by using a balance with an accuracy of ±0.0001 g). Different carriers were used to prepare the spray-dried SB oil, namely, GA, β-CD, and a mixture thereof (GA:β-CD, 1:1). All carriers were purchased from Sigma-Aldrich (Taufkirchen, Germany).

### 2.2. Preparation of the Emulsion

An important step before the encapsulation process of SBO is the preparation of an emulsion. Solutions of GA, β-CD, and a mixture of GA and β-CD (1:1) were prepared by dissolving them in distilled water with constant stirring (800 rpm) at a constant temperature of 50 ± 1 °C overnight to ensure the complete saturation of the polymer molecules. Then, 1 g of the surfactant Tween 20 and SBO were gradually mixed and homogenized at room temperature by using an Ultra-Turrax (model T25, IKA, Staufen, Germany) at 20,000 rpm for 5 min. The ratios of the carrier materials and oil loading were 2:1 (2), 3:1 (3), and 4:1 (4) (*w*/*w*), respectively.

### 2.3. Spray Drying of the Prepared Emulsion

The prepared emulsions were dried by using a laboratory-scale spray dryer, SD 06 (Labplant, North Yorkshire, UK). During the process, the following parameters were kept constant: air flow of 10 m/s, feed flow of 485 mL/h, and deblocking speed at medium level. The corresponding outlet temperatures were 65–85 °C. The experimental design for the SD encapsulation of SBO is shown in Table 1. All SBO powders were prepared in duplicate and stored in opaque, airtight containers in a desiccator at 20 °C until the analysis.

### 2.4. Powder Characterization

#### 2.4.1. Product Yield

The product yield was calculated as the ratio between the dry matter content of the powder obtained after spray drying and the dry matter content of the initial emulsion according to the following equation [22]:

(1) $$Product\;yield\;\left(\%\right)=\frac{m_{p}}{m_{o}+m_{c}}$$

where *m_p_* is the mass (g) of the produced spray-dried SBO powder, *m_o_* is the mass (g) of the oil in the initial emulsion, and *m_c_* is the mass (g) of the carrier in the initial emulsion.

#### 2.4.2. Encapsulation Efficiency

The encapsulation efficiency was determined based on the retention of SBO in the encapsulated powder according to the following equation [23]:

(2) $$EE_{oil}\left(\%\right)=\frac{m_{total\;oil}-m_{surface\;oil}}{m_{total\;oil}}\times 100$$

The total oil content of the SBO powder (5 g) was extracted with *n*-hexane by using a Soxtherm Multistat SX PC extraction device (Gerhardt, Deutschland) according to procedure of Harkat et al. (2022) [24] with minor modifications. The Soxhlet procedure consisted of adding 10 g of SBO powder in a thimble to the Soxhlet extraction system, in which 50 mL of *n*-hexane was refluxed over the sample for 90 min at increasing temperatures (up to 180 °C). The solvent was decanted, and the residue was vacuum-dried on rotavapor (IKA RV10 basic, IKA Werke GmbH & Co. KG, Staufen im Breisgau, Germany) at 70 °C to a constant weight.

The surface oil content was determined by gently shaking the encapsulated powder sample (5 g) in 50 mL of *n*-hexane for 10 min without destroying the microcapsules. The solvent was decanted, and the residue was vacuum-dried at 70 °C to a constant weight.

#### 2.4.3. Moisture Content

The moisture content (%) was calculated by placing the SBO powders in the oven at 105 °C (FN 500; Nüve, Ankara, Turkey) until a constant mass was achieved [25].

#### 2.4.4. Hygroscopicity

The hygroscopicity of the SBO powders was analyzed according to the method of Tonon et al. (2008) [26]. The mass of 1 g of SBO powder was placed in a flat-bottomed weighing bottle in a desiccator containing saturated NaCl solution (RH = 75.3%). After 7 days, the powders were reweighed, and the hygroscopicity (%) was calculated as the water content per 100 g of powder by using the following equation:

(3) $$Hygroscopicity\;\left(g/100g\right)=\frac{m_{7}-m_{1}}{m_{1}}\times 100$$

where *m*_7_ is the mass (g) of the powder after 7 days of storage and m_1_ is the mass (g) of the powder before storage.

#### 2.4.5. Solubility

The solubility of the SBO powders was determined according to the method described by Garofulić et al. (2016) [16]. A mass of 1 g of SBO powder was placed in a Falcon test tube with 10 mL of distilled water and stirred with a vortex vibrator for 1 min, then thermostatted at 37 °C for 30 min in a water bath (B-490; Büchi, Flawil, Switzerland) and centrifuged at 4500× *g* for 15 min (centrifuge Z 206A Hermle, Wehingen, Germany). The obtained supernatant was collected and dried at 105 °C in a laboratory oven (FN 500; Nüve, Ankara, Turkey) until a constant mass was obtained. The solubility (%) was calculated according to the following equation:

(4) $$Solubility\;\left(\%\right)=\frac{m_{s}}{m_{p}}\times 100$$

where *m_s_* is the mass (g) obtained by drying the supernatant and *m_p_* is the mass (g) of the powder used in the analysis.

#### 2.4.6. Bulk Density

The bulk density of the SBO powders was determined according to the method of Koç et al. (2012) [27] with some modifications. An amount of 2 g of powder was weighed into an empty 10 mL graduated cylinder, and the cylinder was held on a vortex vibrator for 1 min to uniformly distribute the powder particles. The cylinder was then placed on a solid and apartment surface, and the volume of the powder was read. The bulk density was calculated as the ratio between the powder mass and the volume occupied in the cylinder (g/mL).

#### 2.4.7. Determination of Total Carotenoids

The total carotenoid content was determined according to the modified method of Chemat et al. (2012) [28]. Powder solutions in hexane (1 g/10 mL (*w*/*v*)) were measured at 460 nm in a spectrophotometer (UV–VIS spectrophotometer Uviline 9400, Secomam, France). The quantification of the carotenoid content was based on calibration with the β-carotene standard, and the total carotenoid amounts were expressed as mg per 100 g dry weight of powder (mg/100 g dm).

#### 2.4.8. Determination of Antioxidant Capacity

The lipophilic ORAC assay was conducted on a 96-well microplate by using a fluorescence plate reader (Clariostar. BMG LABTECH, Offenburg, Germany) according to a method previously described in a study by Zhong et al. (2012) [29]. The reaction consisted of 25 μL of diluted extracts and 150 µL of fluorescein (75 nM), which was used as a target for free-radical attack. The reaction mixtures were incubated for 30 min at 37 °C, the reaction was initiated by the addition of 25 μL of AAPH (240 mM), and the fluorescence was (λ excitation: 485 nm; λ emission: 538 nm) recorded every 90 s for 120 min at 37 °C. The blank was 7% methylated β-CD (*w*/*v*) made in a 50% acetone–water mixture (*v*/*v*). Trolox (25 μL) was used as the standard, and the results were expressed as µmol of Trolox equivalent per g of dried matter (µmol TE/100 g dm).

### 2.5. Experimental Design and Statistical Analysis

The experimental design and statistical analysis were performed by using the STATISTICA v. 10 Experimental design software (DOE) (StatSoft Inc., Tulsa, OK, USA). A three-level full-factorial experimental design was used to determine the influence of the carrier, carrier-to-oil ratio, and inlet air temperature during SD on the physicochemical properties of the SBO powders. The experiments were performed in duplicate, starting with the lowest carrier-to-oil ratio and temperature. Each of the studied parameters was observed at three levels—low (−1), medium (0), and high (1); thus, a total of 27 experiments were included. The levels observed for the carrier-to-oil ratio were 2 (−1), 3 (0), and 4 (1), and those observed for the drying temperature were 120 °C (−1), 150 °C (0), and 180 °C (1). The responses obtained from the experimental design were the product yield (%), encapsulation efficiency (%), moisture content (%), hygroscopicity (g/100 g), solubility (%), bulk density (g/L), total carotenoid content (mg/100 g dm), and antioxidant capacity (μmol TE/100 g dm). The design matrix for the experiment and the regression model for each response were calculated as follows [30]:

$$Y=\beta_{0}+\sum \beta_{i}+\sum \beta_{ii}X_{I}^{2}+\sum \beta_{ij}X_{i}X_{j}$$

where *Y* is the predicted response, *β*_0_ is the fixed response, *β_i_*, *β_ii_*_,_ and *β_ĳ_* are the linear, quadratic, and interaction coefficients, and *X_i_* and *X_j_* are independent factors, respectively. Analysis of variance (ANOVA) was used to determine the significant differences (*p* ≤ 0.5) between the drying conditions that were applied. The model was fitted via multiple linear regressions (MLRs). The validity of the full quadratic empirical model for predicting one dependent variable was tested by using ANOVA at a 95% probability level. A prediction and profiling tool was used for optimization purposes. Preferences were set for each response as follows: For the encapsulation efficiency, solubility, and bulk density, the preference was high (1.0), while for the moisture content and hygroscopicity, it was low (0.0). The factors were adjusted to the optimal values and observed for 20 steps to allow more accurate optimization.

## 3. Results and Discussion

The experimental design for the SD encapsulation of SBO is shown in Table 1, where two carrier materials (GA, β-CD), a mixture thereof, different carrier-to-oil ratios (2–4), and different drying temperatures (120–180 °C) were used. The influence of the following SD parameters on the physicochemical properties of the obtained SBO powders was determined: product yield, encapsulation efficiency, moisture content, hygroscopicity, solubility, and bulk density (Table 2) while the results of ANOVA are summarized in Table 3. Their influence on the total carotenoid content and antioxidant capacity (Table 4) was also determined and the results of ANOVA are shown in Table 5). 

### 3.1. Product Yield

The most important parameter for determining the cost and efficiency of the SD process is the product yield [31]. Oil encapsulation is considered successful when the encapsulated powder has minimal surface oil content and maximal oil retention in the core of the powder particles [6]. The product yield of the obtained SBO powders containing GA as a carrier ranged from 36.79 to 52.18%; it ranged from 49.94 to 64.60% for the powders containing β-CD and from 50.71 to 65.61% for the powders containing a mixture of GA and β-CD (GA:β-CD, 1:1) (Table 2). Regardless of the type of carrier, the lowest product yield was obtained with a carrier-to-oil ratio of 2 at a drying temperature of 120 °C, while the carrier-to-oil ratio of 4 and drying temperature of 180 °C provided the highest product yield, except for the spray-dried powders with GA (150 °C). Among the selected carriers, β-CD and GA:β-CD had very similar and satisfactory product yields of SBO powders (above 50%), indicating a successful SD process [32]. The carrier materials formed a strong three-dimensional protective network around the core material, which inhibited the movement of the droplet matrix and had stronger mechanical properties against particle adhesion in the SD chamber, but fine particle losses could occur through an exhaust air filter during spray drying and during manual manipulation of the powder [33,34,35]. The product yield of encapsulated nettle extracts ranged from 64.63 to 87.23%, and the type and proportion of the carrier significantly affected the product yield [36]. In general, a higher carrier-to-oil ratio and a higher drying temperature during SD had a positive effect on the product yield of the obtained SBO powders. In this study, the highest product yields of the SBO powders were obtained at a carrier-to-oil ratio of 4 and a drying temperature of 180 °C when GA:β-CD (65.61%) and β-CD (64.60%) were used as carriers, as well as at a lower drying temperature of 150 °C when GA (52.18%) was used as a carrier.

The results of the ANOVA showed that the carrier-to-oil ratio and the drying temperature, as well as their combined effect, had a significant influence (*p* < 0.05) on the product yield when GA and β-CD were used as carriers (Table 3). Although the product yield was higher for the powders prepared with GA:β-CD at a higher drying temperature, the temperature did not significantly affect the product yield (Table 2 and Table 3).

This could be due to the greater efficiency of mass and heat transfer at high drying temperatures and the reduction in the retention of drying particles on the wall of the drying chamber [26,37]. The product yield was significantly affected by the drying temperature and oil content when spray drying rice bran oil, and it varied from 45.08 to 73.56% [38]. Similar results were reported by Permal et al. (2020) [39] in a study on optimizing the SD of avocado wastewater. In a study by Correa Filho et al. (2019) [40] on the microencapsulation of β-carotene, an increase in product yield was observed with an increase in GA concentration and drying temperature until a maximum value was reached.

### 3.2. Encapsulation Efficiency

Encapsulation efficiency (EE) is the most important parameter for evaluating the effectiveness of the encapsulation process [41]. It is directly influenced by the characteristics of the carrier and core materials, the properties of the initial emulsion, and the parameters of the spray-drying process, such as the inlet and outlet air temperature, humidity, air flow rate, and the type of atomization [42]. Since the EE is associated with a better core stability and longer shelf-life, it is always desirable to increase the EE by choosing the optimal carrier type and combination of the core-to-carrier ratio with other relevant process variables of drying methods [41]. In this study, the EE in the obtained SBO powders produced with GA ranged from 79.33 to 93.18%; in powders with β-CD, it ranged from 73.08 to 89.13%, and in powders with GA:β-CD, it ranged from to 75.22 to 88.00% (Table 2).

The highest EE in powder containing GA (93.18%) was obtained at a carrier-to-oil ratio of 4 and a drying temperature of 180 °C, while powders containing β-CD and GA:β-CD showed the highest EE at a carrier-to-oil ratio of 3 and a drying temperature of 180 °C for β-CD (89.13%) and 150 °C for GA:β-CD (88%). The carrier-to-oil ratio and inlet air temperature and their combined effect significantly influenced (*p* ≤ 0.05) the EE of the SBO powders (Table 2).

Numerous studies reported that a higher oil load generally resulted in lower EE, and an optimal core-to-wall material ratio of 1:4 was identified for various carriers, such as GA [6]. The stability of the emulsion during SD is also one of the critical parameters for achieving optimal EE [6]. Carriers such as GA increase the viscosity of the aqueous phase of oil-in-water emulsions, which leads to better stabilization of the emulsion and a reduction in circulatory movements within the droplets, resulting in rapid formation of a membrane around the droplets [6].

The results of this study showed that when β-CD and GA:β-CD were used as carriers, the EE of the SBO powders increased when the carrier-to-oil ratio was increased from 2 to 3—or from 3 to 4 in the case of GA—but at higher drying temperatures (Table 2). The higher EE associated with higher carrier-to-oil ratios can be attributed to the smaller amount of core material near the drying surface, which shortens the diffusion path length to the air/particle interface, thus decreasing the surface oil content [6]. A study by Tan et al. (2005) [43] reported that lower oil content in the initial emulsion led to a higher product yield and EE. With an increasing proportion of core material, the EE tended to decrease [44]. Studies by Xu et al. (2020) [23] and Kha et al. (2014) [45] also confirmed that more oil was sufficiently encapsulated at higher carrier-to-oil ratios, which increased the EE of SD.

On the other head, increasing the carrier-to-oil ratio from 3 to 4 when β-CD and GA:β-CD were used as carriers led to a decrease in the encapsulation efficiency of the obtained powders (Table 2). This showed that an optimal amount of carrier was crucial for high EE. A possible explanation is the lower solubility of β-CD in water and the separation of particles at the bottom of the glass, thus reducing the amount of carrier around the core material. Consequently, the larger amount of core material near the drying surface increased the surface oil content and decreased the encapsulation efficiency [6].

The EE was also affected by the drying temperature; according to our results in Table 2, the use of higher drying temperatures resulted in a higher EE of SD. The inlet air temperature was related to the drying rate of the microcapsules and the final water content [46]. The time required to form a hard crust around the droplets was shortened at higher drying temperatures. The crust that was formed had a similar protective effect to that of a semi-permeable membrane and prevented further diffusion of oil droplets to the particle surface, resulting in a high EE [6,23]. The selection of the proper temperature is important because too low of an inlet air temperature can lead to the formation of poor microcapsules that easily clump, have high water content, and have a high membrane density, while too high of an inlet air temperature can cause thermal damage, “ballooning”, excessive bubble growth, and surface defects [6,23]. A similar trend was reported by Aghbashlo et al. (2013) and Xu et al. (2020) [23,47], who observed higher EE at higher drying temperatures when studying the encapsulation of fish oil and sea buckthorn fruit oil, respectively.

### 3.3. Moisture Content

Moisture content is an important indicator of the efficiency and the quality of the final spray-dried product because a lower moisture content can extend the application and acceptability of powders for technological purposes and increase their stability [48]. The moisture content of the spray-dried SBO powders with GA ranged from 0.81 to 3.70%; in the powders with β-CD, it ranged from 0.23 to 2.49%, and in the powders with GA:β-CD, it ranged from 0.79 to 2.79% (Table 2). All obtained powders had moisture contents lower than 4%, and according to Klaypradit and Huang (2008) [49], dry foods with moisture contents between 3 and 10% have good stability during storage.

According to the results of the ANOVA, the carrier-to-oil ratio, drying temperature, and their combined effect significantly (*p* ≤ 0.05) affected the moisture content of the SBO powders (Table 3). Regardless of the carrier used, the lowest moisture content (0.23% for β-CD; 0.79% for GA:β-CD; 0.81% for GA) was determined in the powders at a drying temperature of 180 °C with a carrier-to-oil ratio of 2. Generally, the powders produced with higher carrier-to-oil ratios had higher moisture contents (Table 2). The increase in moisture content in the powders containing higher carrier concentrations was due to the particle size of the carriers, which hindered the diffusion of water during SD [50]. The same trend was observed in the spray-dried powders of rosemary essential oil [50]. Frascareli et al. (2012) [4], who studied the encapsulation of coffee oil, observed that the powders with the lowest moisture content were produced with higher oil concentrations, which could be attributed to the higher hydrophobicity of the obtained powders. According to the results in Table 2, higher drying temperatures had a positive effect on reducing the moisture content in the powders that were obtained. Increasing the drying temperature increased the mass and heat transfer and accelerated the evaporation rate of water, resulting in powders with lower moisture contents [47,51]. Similar results were reported for various powders obtained by SD, such as sea buckthorn fruit pulp oil powder [23], jaboticaba peel extract powder [19], rosemary essential oil powder [47], gac oil powder [52], and lemongrass leaf extract powder [53].

### 3.4. Hygroscopicity

Spray-dried particles can easily absorb moisture in an environment with relatively high humidity, and the surface of the powder becomes sticky, which reduces the quality and shelf-life of dried powders [54].

The hygroscopicity in the SBO powders produced with GA ranged from 1.72 to 6.29 g/100 g; in the powders with β-CD, it ranged from 1.50 to 5.49 g/100 g, and in the powders with GA:β-CD, it ranged from 4.16 to 7.06 g/100 g (Table 2). According to the results of the ANOVA, the carrier-to-oil ratio, inlet air temperature, and their combined effect significantly influenced the hygroscopicity of the SBO powders (Table 3). In general, the results showed that the moisture absorption rate was higher for powders produced at higher drying temperatures and lower carrier-to-oil ratios. The lowest hygroscopicity in powders containing β-CD (1.50 g/100 g) and GA:β-CD (4.16 g/100 g) was determined at 120 °C and a carrier-to-oil ratio of 4. However, in the powders containing GA, the lowest hygroscopicity was obtained at the same carrier-to-oil ratio but at a higher inlet temperature of 180 °C (1.72 g/100 g) (Table 2).

According to Phisuit (2012) [51], an increase in the inlet air temperature led to a reduction in the moisture content in powders, causing the powder to absorb moisture from the environment. Rapid moisture removal during spray drying at higher drying temperatures results in an amorphous product that is in a metastable non-equilibrium state and exhibits a high degree of hygroscopicity [54].

The powders produced with higher carrier-to-oil ratios were less hygroscopic (Table 2), which can be explained by the fact that these powders had higher moisture contents and, consequently, had less of a tendency to absorb moisture from the environment. Powders with lower moisture contents are more hygroscopic, which is related to the larger water concentration gradient between the product and the ambient air [26]. Similar results of decreasing hygroscopicity with increasing carrier concentration and decreasing drying temperature were also found in studies on the encapsulation of rosemary essential oil [55] and acai by SD [26]. Contrary to results of this study, Frascareli et al. (2012) [4] reported higher hygroscopicity in powders produced with higher GA contents due to the hygroscopic nature of GA and the hydrophobic nature of coffee oil.

### 3.5. Solubility

Solubility is considered the most reliable criterion for the behavior of a powder in aqueous solution [56]. The solubility of the obtained SBO powders varied depending on the carrier used for SD (Table 2). As shown in Table 1, the solubility of the SBO powders ranged from 63.70 to 74.70% for the SBO powders containing GA, from 19.55 to 28.00% for the powders containing β-CD, and from 38 to 52% for those containing GA:β-CD. Despite the fact that encapsulation increased the solubility of the oil in water and prevented oil from flowing out to the surface, the solubility of the powders containing β-CD and GA:β-CD was quite low.

Individually, all observed drying parameters had a significant effect on the solubility of the SBO powders (*p* ≤ 0.05). The solubility of the powders containing GA and GA:β-CD was higher for the powders obtained with higher carrier-to-oil ratios. The combined effect of the carrier-to-oil ratio and drying temperature was observed only when GA:β-CD was used as a carrier. The highest solubility was observed at a carrier-to-oil ratio of 4 and a drying temperature of 180 °C for GA (74.70%) and at 150 °C for GA:β-CD (52.00%).

A similar trend of increasing solubility with increasing carrier content and lower oil concentration was observed in studies on the microencapsulation of *Nigella sativa* oil [57] and fish oil [58].

According to Botrel et al. (2014) [58], the solubility of the powder decreased when the initial amount of oil was increased, which could be attributed to the hydrophobic properties of the particles. In our study, the solubility of the β-CD powders was low, and it decreased with increasing β-CD concentration (Table 2), which can be attributed to the lower solubility of β-CD in water. Regarding the drying temperature, the powders produced with GA had higher solubility when dried at higher temperatures, while the powders produced with β-CD and GA:β-CD had higher solubility at lower temperatures. In the study of Elez Garofulić et al. (2016) [18], maltodextrin at 13–17 DE also showed the opposite behavior under the influence of different temperatures when compared to maltodextrin at 4–7 DE and GA, and its solubility decreased at higher drying temperatures.

### 3.6. Bulk Density

The bulk density of powders is an important property from the economic and functional point of view during storage, processing, packaging, and distribution. Low bulk density is not desirable because, in addition to resulting in a larger package volume, it causes the accumulation of a larger amount of air between the particles, thus increasing the possibility of the product’s oxidation and reducing storage stability [59,60]. The bulk density of the powders ranged from 0.37 to 0.44 g/mL in the powders containing GA, from 0.25 to 0.33 g/mL in the powders containing β-CD, and from 0.30 to 0.40 g/mL in the powders containing GA:β-CD (Table 2). According to the results of the ANOVA (Table 3), both the carrier-to-oil ratio and the drying temperature showed a significant (*p* ≤ 0.05) effect on the moisture content of the SBO powders. The highest bulk density in the powders containing GA (0.44 g/mL), GA:β-CD (0.40 g/mL), and β-CD (0.33 g/mL) was determined at 120 °C when the carrier-to-oil ratio was 4. Generally, the powders produced at lower drying temperatures and with higher carrier-to-oil ratios had higher bulk densities. Increasing the temperature of the drying air usually caused a decrease in the apparent density because of a greater tendency to create powders with a greater particle size and a porous and fragmented structure [61,62]. At higher drying temperatures, a hard crust formed around droplets in a short time, which prevented the diffusion of oil droplets to the surface of the particles, resulting in a high EE [6,21]. In a study by Fernandes et al. (2013) [50] on the encapsulation of rosemary essential oil, a higher bulk density was also obtained at lower drying temperatures and higher carrier-to-oil ratios.

### 3.7. Total Carotenoid Content

The total carotenoid content of the powders ranged from 46.09 to 145.01 mg/100 g dm in the powders containing GA, from 79.63 to 277.89 mg/100 g in the powders containing β-CD, and from 56.39 to 176.12 mg/100 g dm in the powders containing GA:β-CD (Table 4). According to the results of the ANOVA, the carrier-to-oil ratio, inlet air temperature, and their combined effect significantly influenced the total carotenoid content of the SBO powders (Table 5). In general, the powders containing GA and GA:β-CD had higher total carotenoid contents at lower carrier-to-oil ratios, while the powders containing β-CD had higher total carotenoid contents at higher carrier-to-oil ratios. This can be explained by the fact that the surrounding walls of CDs are hydrophilic, which allows them to form capsules that serve as hosts for lipophilic compounds and enable the formation of inclusion complexes in their cavities [63,64,65]. Therefore, at higher concentrations of β-CD, more lipophilic molecules have the possibility of forming a complex with the carrier, thus reducing their degradation during drying. On the other hand, increasing the drying temperature generally had a negative effect on the total carotenoid content. The degradation and reduction of the carotenoid content in powders obtained at higher drying temperatures may be caused by the formation of cracks in the surface layer of the powder particles [66]. In addition, higher drying temperatures can damage the surface of the powder particles, which accelerates the release of ingredients from the particles and increases the permeability of gases that affect the degradation of bioactive oil compounds [64]. Our results are in accordance with those of a study by Freitas Santos et al. (2021) [67], wherein a greater retention of carotenoids was observed in powders obtained at lower drying temperatures.

### 3.8. Antioxidant Capacity

One of the most important goals of encapsulation is to stabilize and preserve the biological activity of BAMs, such as tocopherols, fatty acids, and pigments, in oil. The antioxidant capacity in this study was assessed by using an ORAC assay, as previous studies have confirmed that this assay is the most biologically relevant and can measure both lipophilic and hydrophilic antioxidants [68]. The antioxidant capacity of the obtained powders ranged from 897.16 to 1230.42 μmol TE/100 g dm in the powders containing GA, from 871.843 to 1259.88 μmol TE/100 g dm in the powders containing β-CD, and from 887.67 to 1259.88 μmol TE/100 g dm in the powders containing GA:β-CD (Table 4). According to the results of the ANOVA, the carrier-to-oil ratio, inlet air temperature, and their combined effect significantly influenced the antioxidant capacity of the SBO powders (Table 5). In general, the antioxidant capacity of the obtained powders was higher for the powders produced with higher carrier-to-oil ratios at lower drying temperatures. This can be explained by the fact that higher drying temperatures lead to the oxidation of the oil, which increases the peroxide value and decreases the content of antioxidants such as vitamin E [17,69]. According to a study by Selamat et al. (2018) [70], higher carrier-to-oil ratios significantly affected the retention of tocopherol content in a powder obtained by SD and, thus, the retention of antioxidant capacity. Tocopherols form more stable free radicals when compared to unsaturated fatty acids by losing a hydrogen atom from the hydroxyl group, they oxidize faster, and they lead to a more effective antioxidant potential. In addition, they contribute to better oil stability and interrupt chain reactions that increase the formation of harmful free radicals [71]. In a study by Ferreira et al. (2021) [72], a higher antioxidant capacity was also obtained at lower drying temperatures with higher carrier-to-oil ratios.

The physicochemical parameters determined in the SBO powders produced with GA, β-CD, and GA:β-CD were used to predict the response variable values for the desired carrier-to-oil ratio and drying temperature by using regression models (Table 6 and Table 7) and for optimization of SD by using the response surface methodology (RSM), with the aim of obtaining the drying parameters that would result in a powder with a high product yield and EE, high solubility, high bulk density, low moisture content and hygroscopicity (Table 8), and high total carotenoid content and antioxidant capacity (Table 9).

The regression models of the above-mentioned physicochemical parameters were combined with linear, quadratic, and interaction coefficients. The adequacy of the models was tested by calculating the coefficients of determination (R^2^ and R^2^_adj_). R^2^ indicates how much of the observed variability in the data was accounted for by the model, while R^2^_adj_ modifies R^2^ by taking the number of covariates or predictors in the model into account. A well-fitting model should have an R^2^ value of no less than 80% and an R^2^_adj_ value that is close to the R^2^ values, thus ensuring a satisfactory adjustment of the quadratic models to the experimental data. All models had R^2^ values greater than 0.8, and all R^2^_adj_ values were close to those of R^2^, thus confirming the applicability of the models for predicting the physicochemical properties of the SBO powders and implying that the models explained the observed properties of the SBO powders very well (Table 6 and Table 7).

According to the results shown in Table 8, the optimal SD conditions for obtaining powders with desirable physical parameters within the experimental ranges of drying temperatures and carrier-to-oil ratios were: a drying temperature of 162 °C and a carrier-to-oil ratio of 4 when GA was used as a carrier; a drying temperature of 171 °C and a carrier-to-oil ratio of 2.9 when β-CD was used as a carrier; a drying temperature of 131 °C and a carrier-to-oil ratio of 3.2 when GA:β-CD was used as a carrier. The optimal carrier-to-oil ratios were similar when β-CD and GA:β-CD were used for SD (2.9 and 3.2, respectively), whereas the use of GA for the SD of SBO required a greater carrier addition (4). On the other hand, the optimal drying temperatures for the powders with GA and β-CD (162 and 171 °C) were remarkably higher than that for the powders with GA:β-CD (131 °C). These differences were probably the result of the influence of the carriers used. For example, Xu et al. (2020) [23] reported that the optimal conditions for the SD of sea buckthorn pulp oil when using a mixture of GA and maltodextrin were a carrier-to-oil ratio of 5.33 and an inlet air temperature of 154 °C. Roccia et al. (2014) [73] reported that the optimal temperature for the SD of sunflower oil powder when using hydroxypropyl methylcellulose and maltodextrin at DE 15 was 163 °C. In a study by Botrel et al. (2014) [58], the best operating conditions for the spray drying of fish oil when using whey protein and inulin were a drying temperature of 185 °C and the addition of 40% inulin and 6% oil.

Under the optimal conditions, the values for the product yield, EE, moisture content, hygroscopicity, solubility, and bulk density were predicted as 52.36%, 93.13%, 1.73%, 2.40 g/100 g, 73.71%, and 0.41 g/mL in the powders containing GA, as 58.15, 88.00%, 0.25%, 3.22 g/100 g, 22.85%, and 0.27 g/mL in the powders containing β-CD, and as 62.30%, 79.97%, 1.88%, 4.49 g/100 g, 48.24%, and 0.354 g/mL in the powders containing GA:β-CD, respectively. The experimental values of the physical parameters obtained under the optimal conditions were close to the predicted values (Table 8), indicating the suitability of the model for optimizing the SD process for the production of SBO powders.

It can be observed that the powders containing GA had the highest EE, solubility, and bulk density and the lowest hygroscopicity. On the other hand, the powders containing GA:β-CD had the highest product yield, while the powders containing β-CD were characterized by the lowest moisture content.

According to the results shown in Table 9, the optimal drying temperature for obtaining powders with the highest values of total carotenoid content and antioxidant capacity was 120 °C for all carriers used. On the other hand, the optimal carrier-to-oil ratios were lower when using GA and GA:β-CD for SD (2.7 and 3.2, respectively), while the use of β-CD required a higher carrier-to-oil ratio (4). Under the optimal conditions, the values for the total carotenoid content and antioxidant capacity were predicted as 104.21 mg/100 g dm and 1053.28 μmol TE/100 g dm in the powders containing GA, as 277.89 and 1454.39 μmol TE/100 g dm in the powders containing β-CD, and as 126.45 mg/100 g dm and 1121.24 μmol TE/100 g dm in the powders containing GA:β-CD. The experimental values of the chemical parameters obtained under the optimal conditions were close to the predicted values (Table 9), indicating the suitability of the model for the optimization of the SD process for the production of SBO powders. The powders containing β-CD had a significantly higher total carotenoid content and antioxidant capacity than the powders containing GA and GA:β-CD (Table 9).

Considering that the main purpose of SD is to preserve BAMs and their antioxidant capacity, it can be concluded that SD by using β-CD under the SD conditions of a drying temperature of 120 °C and a carrier-to-oil ratio of 4 is the most suitable for the preparation of spray-dried SBO powders with the desirable physicochemical properties. Encapsulation by SD is a good strategy for protecting the oil-sensitive compounds of sea buckthorn from oxidation; the transition from a liquid to a solid state extends its shelf life, and the physical properties of the obtained powder are useful for future product processing. Optimization of the SD of SBO improves the physicochemical properties of powdered products and preserves their bioactive compounds, which enables their wide application in the food industry, e.g., in the production of value-added foods, as well as in the dietary supplement industry.

## 4. Conclusions

Based on the results of the conducted research, it is evident that the parameters of the SD process significantly affect the physicochemical properties of the obtained SBO powders. Higher inlet air drying temperatures and carrier-to-oil ratios increased the product yield, encapsulation efficiency, and solubility. Increasing the carrier-to-oil ratio resulted in a higher bulk density and lower hygroscopicity, while higher drying temperatures decreased the powder moisture content, total carotenoid content, and antioxidant capacity. For the production of the SBO powder with the best physicochemical properties, the following optimal SD process parameters were selected: β-CD as a carrier, a drying temperature of 120 °C, and a carrier-to-oil ratio of 4. This study confirms the need to optimize the SD process to obtain a high-quality product with great potential for application in the food (beverages, dairy, meat, bakery products, soups, etc.) and nutraceutical industries (functional foods and supplements). In order to better understand the behavior and possible applications of the powder, future studies should be focused on the evaluation of powder particles’ morphology, as well as on the sensory properties and storage stability of the obtained spray-dried powders.

## Figures and Tables

**Table 1:** Experimental design for the spray-drying encapsulation of sea buckthorn oil.

Sample	Carrier	Carrier-to-Oil Ratio	Temperature (°C)
1	GA	2	120
2			150
3			180
4		3	120
5			150
6			180
7		4	120
8			150
9			180
10	β-CD	2	120
11			150
12			180
13		3	120
14			150
15			180
16		4	120
17			150
18			180
19	GA:β-CD (1:1)	2	120
20			150
21			180
22		3	120
23			150
24			180
25		4	120
26			150
27			180

**Table 2:** Results of the physical parameters of SBO powders produced by using gum arabic (GA), β-cyclodextrin (β-CD), and a mixture thereof (GA:β-CD) with different carrier-to-oil ratios (2–4) and at different drying temperatures (120–180 °C).

Sample	Product Yield	Encapsulation Efficiency	Moisture Content	Hygroscopicity	Solubility	Bulk Density
	%	%	%	g/100 g	%	g/mL
1	36.79 ± 0.34	79.33 ± 0.56	1.26 ± 0.01	3.65 ± 0.01	63.70 ± 0.55	0.40 ± 0.00
2	37.00 ± 0.21	90.51 ± 0.41	2.18 ± 0.02	4.42 ± 0.01	65.62 ± 0.50	0.42 ± 0.00
3	45.79 ± 0.15	88.26 ± 0.49	3.70 ± 0.01	2.77 ± 0.02	68.59 ± 0.51	0.44 ± 0.01
4	42.28 ± 0.25	87.06 ± 0.85	1.05 ± 0.02	3.99 ± 0.03	63.82 ± 0.62	0.38 ± 0.01
5	50.91 ± 0.31	90.62 ± 0.65	2.12 ± 0.02	3.28 ± 0.03	68.69 ± 0.58	0.40 ± 0.00
6	52.18 ± 0.33	92.42 ± 0.68	2.03 ± 0.03	2.68 ± 0.02	72.65 ± 0.60	0.42 ± 0.01
7	46.62 ± 0.18	91.76 ± 0.52	0.81 ± 0.01	6.29 ± 0.04	65.41 ± 0.62	0.37 ± 0.00
8	45.87 ± 0.25	91.71 ± 0.54	1.73 ± 0.02	3.19 ± 0.03	73.23 ± 0.68	0.37 ± 0.00
9	50.09 ± 0.34	93.18 ± 0.50	1.69 ± 0.01	1.72 ± 0.01	74.70 ± 0.64	0.39 ± 0.00
10	49.94 ± 0.33	83.67 ± 0.64	1.59 ± 0.01	2.45 ± 0.02	26.00 ± 0.15	0.30 ± 0.01
11	55.29 ± 0.25	84.17 ± 0.60	2.17 ± 0.02	2.18 ± 0.01	23.00 ± 0.21	0.31 ± 0.00
12	62.48 ± 0.22	73.08 ± 0.62	2.49 ± 0.01	1.50 ± 0.00	21.00 ± 0.19	0.33 ± 0.01
13	57.73 ± 0.45	83.97 ± 0.57	0.23 ± 0.01	5.49 ± 0.04	24.00 ± 0.20	0.28 ± 0.00
14	54.44 ± 0.36	85.64 ± 0.52	0.32 ± 0.00	3.02 ± 0.02	21.00 ± 0.15	0.29 ± 0.00
15	59.14 ± 0.33	80.18 ± 0.59	0.87 ± 0.01	4.02 ± 0.02	19.55 ± 0.13	0.30 ± 0.01
16	60.44 ± 0.33	83.63 ± 0.62	0.40 ± 0.01	4.42 ± 0.03	28.00 ± 0.18	0.25 ± 0.00
17	60.94 ± 0.36	89.13 ± 0.67	0.56 ± 0.01	3.13 ± 0.02	24.00 ± 0.17	0.26 ± 0.01
18	64.60 ± 0.38	78.82 ± 0.52	0.60 ± 0.02	4.61 ± 0.02	22.00 ± 0.18	0.28 ± 0.01
19	50.71 ± 0.25	79.23 ± 0.45	2.05 ± 0.03	5.24 ± 0.02	48.00 ± 0.20	0.34 ± 0.01
20	57.19 ± 0.24	82.62 ± 0.51	2.50 ± 0.02	4.71 ± 0.03	50.00 ± 0.19	0.34 ± 0.01
21	62.34 ± 0.38	75.22 ± 0.50	2.79 ± 0.02	4.16 ± 0.03	52.00 ± 0.22	0.40 ± 0.01
22	54.56 ± 0.34	83.49 ± 0.62	1.49 ± 0.02	6.31 ± 0.04	43.00 ± 0.35	0.32 ± 0.00
23	59.62 ± 0.32	88.00 ± 0.45	1.07 ± 0.02	4.76 ± 0.03	45.00 ± 0.31	0.34 ± 0.00
24	63,41 ± 0.36	76.09 ± 0.68	1.81 ± 0.01	5.86 ± 0.04	49.00 ± 0.30	0.36 ± 0.00
25	55.55 ± 0.45	80.18 ± 0.72	0.79 ± 0.02	7.06 ± 0.05	38.00 ± 0.25	0.30 ± 0.01
26	60.48 ± 0.39	85.56 ± 0.78	1.02 ± 0.02	6.64 ± 0.04	44.00 ± 0.30	0.31 ± 0.00
27	65.61 ± 0.25	79.55 ± 0.70	1.56 ± 0.02	5.21 ± 0.04	44.00 ± 0.22	0.33 ± 0.00

Results are expressed as the mean ± standard deviation (SD).

**Table 3:** Analysis of variance (ANOVA) for the effects of the temperature (X_1_) and carrier-to-oil ratio (X_2_) on the observed physical parameters of SBO powders produced with gum arabic (GA), β-cyclodextrin (β-CD), and a mixture thereof (GA:β-CD) at the 95% confidence level.

Carrier	Source of Variation	Product Yield		Encapsulation Efficiency		Moisture Content		Hygroscopicity		Solubility		Bulk Density	
		F-Ratio	*p*-Value	F-Ratio	*p*-Value	F-Ratio	*p*-Value	F-Ratio	*p*-Value	F-Ratio	*p*-Value	F-Ratio	*p*-Value
GA	X_1_	50.37	*p* < 0.01	25.64	*p* < 0.01	3327.28	*p* < 0.01	213.77	*p* < 0.01	16.41	*p* < 0.01	18.90	*p* < 0.01
	X_2_	32.26	*p* < 0.01	22.41	*p* < 0.01	7240.24	*p* < 0.01	5918.49	*p* < 0.01	37.38	*p* < 0.01	17.34	*p* < 0.01
	X_1_X_2_	6.68	0.01	7.55	0.01	1228.75	*p* < 0.01	2116.88	*p* < 0.01	2.22	0.15	1.67	0.24
β-CD	X_1_	66.93	*p* < 0.01	9.49	0.01	1676.33	*p* < 0.01	11,146.59	*p* < 0.01	25.97	*p* < 0.01	35.84	*p* < 0.01
	X_2_	68.59	*p* < 0.01	55.82	*p* < 0.01	169.28	*p* < 0.01	3528.58	*p* < 0.01	70.87	*p* < 0.01	12.15	*p* < 0.01
	X_1_X_2_	19.90	*p* < 0.01	4.32	0.03	28.62	*p* < 0.01	1392.99	*p* < 0.01	0.61	0.67	0.33	0.85
GA:β-CD (1:1)	X_1_	3.84	0.06	21.56	*p* < 0.01	10,781.50	*p* < 0.01	2523.17	*p* < 0.01	156.66	*p* < 0.01	29.78	*p* < 0.01
	X_2_	127.38	*p* < 0.01	95.86	*p* < 0.01	2501.44	*p* < 0.01	1329.92	*p* < 0.01	70.91	*p* < 0.01	25.96	*p* < 0.01
	X_1_X_2_	8.06	*p* < 0.01	21.99	*p* < 0.01	395.41	*p* < 0.01	549.17	*p* < 0.01	5.79	0.01	2.71	0.10

Effect of temperature (X_1_); effect of carrier-to-oil ratio (X_2_).

**Table 4:** Results for the total carotenoid content and antioxidant capacity of SBO powders produced by using gum arabic (GA), β-cyclodextrin (β-CD), and a mixture thereof (MIX) with different carrier-to-oil ratios (2–4) and at different drying temperatures (120–180 °C).

Sample	Total Carotenoids	ORAC
	mg/100 g dm	μmol TE/100 g dm
1	129.95 ± 0.56	996.99 ± 1.56
2	93.24 ± 0.28	1085.78 ± 1.78
3	56.94 ± 0.23	1230.42 ± 1.49
4	104.89 ± 0.65	959.47 ± 1.23
5	62.42 ± 0.41	1068.05 ± 1.95
6	56.33 ± 0.38	1196.25 ± 2.15
7	145.01 ± 0.64	897.16 ± 1.58
8	46.09 ± 0.41	1000.15 ± 1.49
9	53.10 ± 0.29	1095.20 ± 1.78
10	185.21 ± 0.69	883.81 ± 0.97
11	179.80 ± 0.72	1189.80 ± 1.97
12	277.89 ± 0.85	1454.39 ± 2.12
13	149.21 ± 0.56	871.83 ± 1.18
14	79.63 ± 0.28	1169.92 ± 1.17
15	209.70 ± 0.79	1292.47 ± 2.26
16	189.01 ± 0.56	927.98 ± 1.78
17	220.51 ± 0.87	1201.97 ± 1.97
18	217.09 ± 0.79	1278.44 ± 2.11
19	176.12 ± 0.56	939.37 ± 1.87
20	136.85 ± 0.74	1088.76 ± 1.47
21	76.31 ± 0.35	1259.88 ± 2.47
22	123.84 ± 0.48	892.45 ± 0.79
23	59.92 ± 0.25	887.67 ± 0.59
24	56.39 ± 0.38	1154.31 ± 1.42
25	153.89 ± 0.54	939.25 ± 0.93
26	109.68 ± 0.47	1032.95 ± 1.18
27	71.27 ± 0.36	1194.95 ± 1.89

Results are expressed as the mean ± standard deviation (SD).

**Table 5:** Analysis of variance (ANOVA) for the effects of the temperature (X_1_) and carrier-to-oil ratio (X_2_) on the observed chemical parameters (total carotenoid content and antioxidant capacity) of SBO powder produced with gum arabic (GA), β-cyclodextrin (β-CD), and a mixture thereof (GA:β-CD) at the 95% confidence level.

Carrier	Source of Variation	Total Carotenoids		ORAC	
		F-Ratio	*p*-Value	F-Ratio	*p*-Value
GA	X_1_	79.93	*p* < 0.01	316.99	*p* < 0.01
	X_2_	1279.26	*p* < 0.01	1296.71	*p* < 0.01
	X_1_X_2_	104.91	*p* < 0.01	6.96	0.01
β-CD	X_1_	228.08	*p* < 0.01	130.29	*p* < 0.01
	X_2_	251.24	*p* < 0.01	6328.57	*p* < 0.01
	X_1_X_2_	75.30	*p* < 0.01	153.57	*p* < 0.01
GA:β-CD (1:1)	X_1_	321.61	*p* < 0.01	339.88	*p* < 0.01
	X_2_	890.94	*p* < 0.01	1962.33	*p* < 0.01
	X_1_X_2_	35.53	*p* < 0.01	56.47	*p* < 0.01

Results are expressed as the mean ± standard deviation (SD).

**Table 6:** Regression models for the physical parameters of the spray-dried SBO powders produced with the addition of gum arabic (GA), β-cyclodextrin (β-CD), and a mixture thereof (GA:β-CD) and the corresponding values of the coefficient of determination (R^2^) and adjusted coefficient of determination (R^2^_adj_).

Carrier	Response	Model	R^2^	R^2^_adj_
GA	Product yield	1492.24 − 19.87X_1_ + 0.07X_1_^2^ − 1099.64X_2_ + 177.60X_2_^2^ + 14.987X_1_X_2_ − 2.39X_1_X_2_^2^ − 0.05X_1_^2^X_2_ − 0.01X_1_^2^X_2_^2^	0.955	0.915
	Encapsulation efficiency	−574.946 + 7.331X_1_ − 0.020X_1_^2^ + 440.033X_2_ − 71.714X_2_^2^ − 4.950X_1_X_2_ + 0.82X_1_X_2_^2^ + 0.014X_1_^2^X_2_ − 0.002X_1_^2^X_2_^2^	0.933	0.874
	Moisture content	90.2036 − 1.1783X_1_ − 0.013X_1_^2^ + 210.324X_2_ − 37.047X_2_^2^ − 2.957X_1_X_2_ + 0.520X_1_X_2_^2^ + 0.010X_1_^2^X_2_ − 0.002X_1_^2^X_2_^2^	0.999	0.999
	Hygroscopicity	−41.21 + 0.2505X_1_ + 0.0036X_1_^2^ − 71.7703X_2_ + 13.7842X_2_^2^ + 0.9449X_1_X_2_ − 0.1785X_1_X_2_^2^ − 0.0029X_1_^2^X_2_ + 0.0006X_1_^2^X_2_^2^	0.999	0.999
	Solubility	21.0877 + 0.9418X_1_ − 0.0049X_1_^2^ + 53.953X_2_ − 12.7781X_2_^2^ − 1.0307X_1_X_2_ + 0.2259X_1_X_2_^2^ + 0.0048X_1_^2^X_2_ − 0.001X_1_^2^X_2_^2^	0.928	0.864
	Bulk density	4.76827 − 0.0582X_1_ + 00.00019X_1_^2^ − 3.096X_2_ + 0.50305X_2_^2^ + 0.04118X_1_X_2_ − 0.00662X_1_X_2_^2^ − 0.00014X_1_^2^X_2_ − 0.00002X_1_^2^X_2_^2^	0.898	0.807
β-CD	Product yield	−758.312 + 10.885X_1_ − 0.035X_1_^2^ + 495.65X_2_ − 66.272X_2_^2^ − 6.783X_1_X_2_ + 0.926X_1_X_2_^2^ + 0.022X_1_^2^X_2_ − 0.03X_1_^2^X_2_^2^	0.975	0.953
	Encapsulation efficiency	−451.067 + 7.182X_1_ − 0.025X_1_^2^ + 424.259X_2_ − 80.369X_2_^2^ − 5.655X_1_X_2_ + 1.059X_1_X_2_^2^ + 0.02X_1_^2^X_2_ − 0.004X_1_^2^X_2_^2^	0.943	0.892
	Moisture content	−42.1108 + 0.58X_1_ − 0.0019X_1_^2^ + 48.435X_2_ − 30.892X_2_^2^ − 2.623X_1_X_2_ + 0.409X_1_X_2_^2^ + 0.008X_1_^2^X_2_ − 0.001X_1_^2^X_2_^2^	0.998	0.996
	Hygroscopicity	−320.9189 + 4.331X_1_ + 0.014X_1_^2^ + 196.808X_2_ − 30.892X_2_^2^ − 2.623X_1_X_2_ + 0.409X_1_X_2_^2^ + 0.008X_1_^2^X_2_ − 0.001X_1_^2^X_2_^2^	0.999	0.999
	Solubility	113.4 − 1.1667X_1_ + 0.043X_1_^2^ − 7.5X_2_ − 1.1X_2_^2^ + 0.0667X_1_X_2_ + 0.0167X_1_X_2_^2^ − 0.0004X_1_^2^X_2_	0.956	0.917
	Bulk density	2.309 − 0.027X_1_ + 0.00009X_1_^2^ − 1.54219X_2_ + 0.27427X_2_^2^ + 0.02080X_1_X_2_ − 0.00367X_1_X_2_^2^ − 0.00007X_1_^2^X_2_ + 0.00001X_1_^2^X_2_^2^	0.915	0.840
GA:β-CD (1:1)	Product yield	−744.076 + 9.434X_1_ − 0.028X_1_^2^ + 547.363X_2_ − 85.999X_2_^2^ − 6.484X_1_X_2_ + 1.023X_1_X_2_^2^ + 0.019X_1_^2^X_2_ − 0.003X_1_^2^X_2_^2^	0.970	0.944
	Encapsulation efficiency	−1186.34 + 17.09X_1_ − 0.06X_1_^2^ + 851.83X_2_ − 132.71X_2_^2^ − 11.45X_1_X_2_ + 1.77X_1_X_2_^2^ + 0.04X_1_^2^X_2_ − 0.01X_1_^2^X_2_^2^	0.973	0.949
	Moisture content	−115.313 + 1.605X_1_ − 0.005X_1_^2^ + 86.044X_2_ − 13.433X_2_^2^ − 1.1687X_1_X_2_ + 0.183X_1_X_2_^2^ + 0.004X_1_^2^X_2_ − 0.001X_1_^2^X_2_^2^	0.999	0.999
	Hygroscopicity	−274.701 + 3.918X_1_ − 0.013X_1_^2^ + 210.324X_2_ − 37.047X_2_^2^ − 2.957X_1_X_2_ + 0.520X_1_X_2_^2^ + 0.010X_1_^2^X_2_ − 0.002X_1_^2^X_2_^2^	0.999	0.998
	Solubility	−340.00 + 5.733X_1_ − 0.021X_1_^2^ + 312.00X_2_ − 54.00X_2_^2^ − 4.517X_1_X_2_ + 0.783X_1_X_2_^2^ + 0.016X_1_^2^X_2_ − 0.003X_1_^2^X_2_^2^	0.981	0.965
	Bulk density	3.07074 − 0.03441X_1_ + 0.00011X_1_^2^ − 2.16547X_2_ + 0.39498X_2_^2^ + 0.2738X_1_X_2_ − 0.00493X_1_X_2_^2^ − 0.00009X_1_^2^X_2_ + 0.0002X_1_^2^X_2_^2^	0.931	0.871

Effect of temperature (X_1_); effect of the carrier-to-oil ratio (X_2_).

**Table 7:** Regression models for the chemical parameters (total carotenoid content and antioxidant capacity) of the spray-dried SBO powders produced with the addition of gum arabic (GA), β-cyclodextrin (β-CD), and a mixture thereof (GA:β-CD) and the corresponding values of the coefficient of determination (R^2^) and adjusted coefficient of determination (R^2^_adj_).

Carrier	Response	Model	R^2^	R^2^_adj_
GA	Total carotenoids	2500.56 − 36.96X_1_ + 0.15X_1_^2^ − 1001.72X_2_ + 96.23X_2_^2^ + 17.01X_1_X_2_ − 1.92X_1_X_2_^2^ − 0.07X_1_^2^X_2_ + 0.01X_1_^2^X_2_^2^	0.997	0.995
	ORAC	2249.89 − 13.985X_1_ + 0.029X_1_^2^ − 962.584X_2_ + 143.702X_2_^2^ + 10.734X_1_X_2_ − 1.254X_1_X_2_^2^ − 0.026X_1_^2^X_2_ + 0.002X_1_^2^X_2_^2^	0.997	0.995
β-CD	Total carotenoids	−13485.8 + 199.7X_1_ − 0.7X_1_^2^ + 10875.1X_2_ − 1794.3X_2_^2^ − 159X_1_X_2_ + 26.4X_1_X_2_^2^ + 0.6X_1_^2^X_2_ − 0.1X_1_^2^X_2_^2^	0.993	0.987
	ORAC	6113.31 − 78.85X_1_ + 0.24X_1_^2^ − 3867.04X_2_ + 808.49X_2_^2^ + 55.41X_1_X_2_ − 10.65X_1_X_2_^2^ − 0.16X_1_^2^X_2_ + 0.03X_1_^2^X_2_^2^	0.999	0.999
GA:β-CD (1:1)	Total carotenoids	−4930.09 + 70.36X_1_ − 0.23X_1_^2^ + 4780.04X_2_ − 855.38X_2_^2^ − 65.39X_1_X_2_ + 11.58X_1_X_2_^2^ + 0.21X_1_^2^X_2_ − 0.04X_1_^2^X_2_^2^	0.997	0.993
	ORAC	−21035.1 + 299.2X_1_ − 1X_1_^2^ + 17054.6X_2_ − 2752.4X_2_^2^ − 233.6X_1_X_2_ + 38.1X_1_X_2_^2^ + 0.8X_1_^2^X_2_ − 0.1X_1_^2^X_2_^2^	0.998	0.996

Effect of temperature (X_1_); effect of the carrier-to-oil ratio (X_2_).

**Table 8:** Predicted and experimental values of the physical parameters of the spray-dried SBO powder produced with the addition of gum arabic (GA), β-cyclodextrin (β-CD), and a mixture thereof (GA:β-CD) at optimal conditions for each carrier used.

Carrier	Optimal Drying Conditions			Product Yield	Encapsulation Efficiency	Moisture Content	Hygroscopicity	Solubility	Bulk Density
	Temperature/°C	Carrier-to-Oil Ratio		%	%	%	g/100 g	%	g/mL
GA	162	4	Predicted value	52.36	93.13	1.73	2.40	73.71	0.41
			Experimental value	53.12	92.45	1.68	2.37	75.18	0.40
β-CD	171	2.9	Predicted value	58.15	88.00	0.25	3.22	22.85	0.27
			Experimental value	59.42	87.55	0.24	3.20	23.15	0.25
GA:β-CD (1:1)	131	3.2	Predicted value	62.30	79.97	1.88	4.49	48.24	0.35
			Experimental value	63.15	80.25	1.92	4.52	49.26	0.33

**Table 9:** Predicted and experimental values of the chemical parameters (total carotenoid content and antioxidant capacity) of the spray-dried SBO powders produced with the addition of gum arabic (GA), β-cyclodextrin (β-CD), and a mixture thereof (GA:β-CD) at optimal conditions for each carrier used.

Carrier	Optimal Drying Conditions			Total Carotenoids	ORAC
	Temperature/°C	Carrier-to-Oil Ratio		mg/100 g dm	μmol TE/100 g dm
GA	120	2.7	Predicted value	104.21	1053.28
			Experimental value	102.98	1050.75
β-CD	120	4	Predicted value	277.89	1454.39
			Experimental value	275.14	1451.28
GA:β-CD (1:1)	120	3.2	Predicted value	126.45	1121.24
			Experimental value	124.89	1119.96

## Data Availability

The data presented in this study are available on request from the corresponding author.

## References

[1] Pinto D., Franco S.D., Silva A.M., Cupara S., Koskovac M., Kojicic K., Soares S., Rodrigues F., Sut S., Dall’Acqua S. (2019). Chemical characterization and bioactive properties of a coffee-like beverage prepared from Quercus cerris kernels. Food Funct..

[2] Wang Z., Zhao F.L., Wei P.P., Chai X.Y., Hou G.G., Meng Q.G. (2022). Phytochemistry, health benefits, and food applications of sea buckthorn (*Hippophae rhamnoides* L.): A comprehensive review. Front. Nutr..

[3] Mohammed N.K., Tan C.P., Manap Y.A., Muhialdin B.J., Hussin A.S.M. (2020). Spray Drying for the Encapsulation of Oils-A Review. Molecules.

[4] Frascareli E.C., Silva V.M., Tonon R.V., Hubinger M.D. (2012). Effect of process conditions on the microencapsulation of coffee oil by spray drying. Food Bioprod. Process..

[5] Sartomo A., Santoso B., Ubaidillah, Muraza O. (2020). Recent progress on mixing technology for water-emulsion fuel: A review. Energy Convers. Manag..

[6] Jafari S.M., Assadpoor E., He Y.H., Bhandari B. (2008). Encapsulation efficiency of food flavours and oils during spray drying. Dry Technol..

[7] Shishir M.R.I., Chen W. (2017). Trends of spray drying: A critical review on drying of fruit and vegetable juices. Trends Food Sci. Technol..

[8] Gomez-Aldapa C.A., Castro-Rosas J., Rangel-Vargas E., Navarro-Cortez R.O., Cabrera-Canales Z.E., Diaz-Batalla L., Martinez-Bustos F., Guzman-Ortiz F.A., Falfan-Cortes R.N. (2019). A modified Achira (*Canna indica* L.) starch as a wall material for the encapsulation of Hibiscus sabdariffa extract using spray drying. Food Res. Int..

[9] Anandharamakrishnan C., Ishwarya S.P. (2015). Spray Drying Techniques for Food Ingredient Encapsulation Preface.

[10] Turchiuli C., Munguia M.T.J., Sanchez M.H., Ferre H.C., Dumoulin E. (2014). Use of different supports for oil encapsulation in powder by spray drying. Powder Technol..

[11] Klein M., Aserin A., Ben Ishai P., Garti N. (2010). Interactions between whey protein isolate and gum Arabic. Colloids Surf. B. Biointerfaces.

[12] Watson M.A., Lea J.M., Bett-Garber K.L. (2017). Spray drying of pomegranate juice using maltodextrin/cyclodextrin blends as the wall material. Food Sci. Nutr..

[13] Szente L., Szejtli J. (2004). Cyclodextrins as food ingredients. Trends Food Sci. Technol..

[14] Singh M., Sharma R., Banerjee U.C. (2002). Biotechnological applications of cyclodextrins. Biotechnol. Adv..

[15] Tonon R.V., Freitas S.S., Hubinger M.D. (2011). Spray Drying of Acai (*Euterpe oleraceae* Mart.) Juice: Effect of Inlet Air Temperature and Type of Carrier Agent. J. Food Process. Preserv..

[16] Gawalek J., Domian E., Ryniecki A., Bakier S. (2017). Effects of the spray drying conditions of chokeberry (*Aronia melanocarpa* L.) juice concentrate on the physicochemical properties of powders. Int. J. Food Sci. Technol..

[17] Tonon R.V., Grosso C.R.F., Hubinger M.D. (2011). Influence of emulsion composition and inlet air temperature on the microencapsulation of flaxseed oil by spray drying. Food Res. Int..

[18] Garofulic I.E., Zoric Z., Pedisic S., Dragovic-Uzelac V. (2016). Optimization of Sour Cherry Juice Spray Drying as Affected by Carrier Material and Temperature. Food Technol. Biotechnol..

[19] Silva P.I., Stringheta P.C., Teofilo R.F., de Oliveira I.R.N. (2013). Parameter optimization for spray-drying microencapsulation of jaboticaba (*Myrciaria jaboticaba*) peel extracts using simultaneous analysis of responses. J. Food Eng..

[20] Tan S.P., Kha T.C., Parks S.E., Stathopoulos C.E., Roach P.D. (2015). Effects of the spray-drying temperatures on the physiochemical properties of an encapsulated bitter melon aqueous extract powder. Powder Technol..

[21] Asofiei I., Calinescu I., Trifan A., David I.G., Gavrila A.I. (2016). Microwave-Assisted Batch Extraction of Polyphenols from Sea Buckthorn Leaves. Chem. Eng. Commun..

[22] Zhang L.Q., Zeng X.H., Fu N., Tang X., Sun Y., Lin L. (2018). Maltodextrin: A consummate carrier for spray-drying of xylooligosaccharides. Food Res. Int..

[23] Xu S.N., Tang Z.S., Liu H.B., Wang M., Sun J., Song Z.X., Cui C.L., Sun C., Liu S.J., Wang Z. (2020). Microencapsulation of sea buckthorn (*Hippophae rhamnoides* L.) pulp oil by spray drying. Food Sci. Nutr..

[24] Harkat H., Bousba R., Benincasa C., Atrouz K., Gueltekin-oezgueven M., Altuntas U., Demircan E., Zahran H.A., Oezcelik B. (2022). Assessment of Biochemical Composition and Antioxidant Properties of Algerian Date Palm (*Phoenix dactylifera* L.) Seed Oil. Plants.

[25] Azmir J., Zaidul I.S.M., Rahman M.M., Sharif K.M., Mohamed A., Sahena F., Jahurul M.H.A., Ghafoor K., Norulaini N.A.N., Omar A.K.M. (2013). Techniques for extraction of bioactive compounds from plant materials: A review. J. Food Eng..

[26] Tonon R.V., Brabet C., Hubinger M.D. (2008). Influence of process conditions on the physicochemical properties of acai (*Euterpe oleraceae* Mart.) powder produced by spray drying. J. Food Eng..

[27] Koc B., Sakin-Yilmazer M., Kaymak-Ertekin F., Balkir P. (2014). Physical properties of yoghurt powder produced by spray drying. J. Food Sci. Technol..

[28] Gao X.Q., Ohlander M., Jeppsson N., Bjork L., Trajkovski V. (2000). Changes in antioxidant effects and their relationship to phytonutrients in fruits of sea buckthorn (*Hippophae rhamnoides* L.) during maturation. J. Agric. Food Chem..

[29] Zhong Y., Ma C.M., Shahidi F. (2012). Antioxidant and antiviral activities of lipophilic epigallocatechin gallate (EGCG) derivatives. J. Funct. Foods.

[30] Barboni T., Cannac M., Massi L., Perez-Ramirez Y., Chiaramonti N. (2010). Variability of Polyphenol Compounds in *Myrtus communis* L. (Myrtaceae) Berries from Corsica. Molecules.

[31] Muzaffar K., Kumar P. (2015). Parameter optimization for spray drying of tamarind pulp using response surface methodology. Powder Technol..

[32] Bhandari B.R., Datta N., Howes T. (1997). Problems associated with spray drying of sugar-rich foods. Dry Technol..

[33] Fang Z.X., Bhandari B. (2011). Effect of spray drying and storage on the stability of bayberry polyphenols. Food Chem..

[34] Barros L., Alves C.T., Duenas M., Silva S., Oliveira R., Carvalho A.M., Henriques M., Santos-Buelga C., Ferreira I.C.F.R. (2013). Characterization of phenolic compounds in wild medicinal flowers from Portugal by HPLC-DAD-ESI/MS and evaluation of antifungal properties. Ind. Crop. Prod..

[35] Cai Z., Qu Z.Q., Lan Y., Zhao S.J., Ma X.H., Wan Q., Jing P., Li P.F. (2016). Conventional, ultrasound-assisted, and accelerated-solvent extractions of anthocyanins from purple sweet potatoes. Food Chem..

[36] Cegledi E., Garofulic I.E., Zoric Z., Roje M., Dragovic-Uzelac V. (2022). Effect of Spray Drying Encapsulation on Nettle Leaf Extract Powder Properties, Polyphenols and Their Bioavailability. Foods.

[37] Fazaeli M., Emam-Djomeh Z., Ashtari A.K., Omid M. (2012). Effect of spray drying conditions and feed composition on the physical properties of black mulberry juice powder. Food Bioprod. Process..

[38] Vallverdu-Queralt A., Regueiro J., Martinez-Huelamo M., Alvarenga J.F.R., Leal L.N., Lamuela-Raventos R.M. (2014). A comprehensive study on the phenolic profile of widely used culinary herbs and spices: Rosemary, thyme, oregano, cinnamon, cumin and bay. Food Chem..

[39] Permal R., Chang W.L., Chen T., Seale B., Hamid N., Kam R. (2020). Optimising the Spray Drying of Avocado Wastewater and Use of the Powder as a Food Preservative for Preventing Lipid Peroxidation. Foods.

[40] Correa L.C., Lourenco M.M., Moldao-Martins M., Alves V.D. (2019). Microencapsulation of beta-Carotene by Spray Drying: Effect of Wall Material Concentration and Drying Inlet Temperature. Int. J. Food Sci..

[41] Carocho M., Barros L., Bento A., Santos-Buelga C., Morales P., Ferreira I.C.F.R. (2014). *Castanea sativa* Mill. Flowers amongst the Most Powerful Antioxidant Matrices: A Phytochemical Approach in Decoctions and Infusions. Biomed. Res. Int..

[42] Saponjac V.T., Canadanovic-Brunet J., Cetkovic G., Jakisic M., Djilas S., Vulic J., Stajcic S. (2016). Encapsulation of Beetroot Pomace Extract: RSM Optimization, Storage and Gastrointestinal Stability. Molecules.

[43] Tan L.H., Chan L.W., Heng P.W.S. (2005). Effect of oil loading on microspheres produced by spray drying. J. Microencapsul..

[44] Choudhury N., Meghwal M., Das K. (2021). Microencapsulation: An overview on concepts, methods, properties and applications in foods. Food Front..

[45] Kha T.C., Nguyen M.H., Roach P.D. (2010). Effects of spray drying conditions on the physicochemical and antioxidant properties of the Gac (*Momordica cochinchinensis*) fruit aril powder. J. Food Eng..

[46] Kaderides K., Goula A.M., Adamopoulos K.G. (2015). A process for turning pomegranate peels into a valuable food ingredient using ultrasound-assisted extraction and encapsulation. Innov. Food Sci. Emerg..

[47] Aghbashlo M., Mobli H., Rafiee S., Madadlou A. (2013). A review on exergy analysis of drying processes and systems. Renew. Sustain. Energy Rev..

[48] Rattes A.L.R., Oliveira W.P. (2007). Spray drying conditions and encapsulating composition effects on formation and properties of sodium diclofenac microparticles. Powder Technol..

[49] Klaypradit W., Huang Y.W. (2008). Fish oil encapsulation with chitosan using ultrasonic atomizer. LWT Food Sci. Technol..

[50] Fernandes R.V.D., Borges S.V., Botrel D.A. (2013). Influence of spray drying operating conditions on microencapsulated rosemary essential oil properties. Food Sci. Technol..

[51] Chaves J.O., de Souza M.C., da Silva L.C., Lachos-Perez D., Torres-Mayanga P.C., Machado A.P.D., Forster-Carneiro T., Vazquez-Espinosa M., Gonzalez-de-Peredo A.V., Barbero G.F. (2020). Extraction of Flavonoids From Natural Sources Using Modern Techniques. Front. Chem..

[52] Kha T.C., Nguyen M.H., Roach P.D., Stathopoulos C.E. (2014). Microencapsulation of Gac oil: Optimisation of spray drying conditions using response surface methodology. Powder Technol..

[53] Tran T.T.A., Nguyen H.V.H. (2018). Effects of Spray-Drying Temperatures and Carriers on Physical and Antioxidant Properties of Lemongrass Leaf Extract Powder. Beverages.

[54] March R.E., Miao X.S. (2004). A fragmentation study of kaempferol using electrospray quadrupole time-of-flight mass spectrometry at high mass resolution. Int. J. Mass Spectrom..

[55] Fernandes R.V.D., Borges S.V., Botrel D.A., de Oliveira C.R. (2014). Physical and chemical properties of encapsulated rosemary essential oil by spray drying using whey protein-inulin blends as carriers. Int. J. Food Sci. Technol..

[56] Chen X.D., Patel K.C. (2008). Manufacturing Better Quality Food Powders from Spray Drying and Subsequent Treatments. Dry Technol..

[57] Mohammed N.K., Tan C.P., Abd Manap Y., Alhelli A.M., Hussin A.S.M. (2017). Process conditions of spray drying microencapsulation of Nigella sativa oil. Powder Technol..

[58] Botrel D.A., Borges S.V., Fernandes R.V.D., do Carmo E.L. (2014). Optimization of Fish Oil Spray Drying Using a Protein:Inulin System. Dry Technol..

[59] Goula A.M., Adamopoulos K.G. (2004). Spray drying of tomato pulp: Effect of feed concentration. Dry Technol..

[60] Kurozawa L.E., Morassi A.G., Vanzo A.A., Park K.J., Hubinger M.D. (2009). Influence of Spray Drying Conditions on Physicochemical Properties of Chicken Meat Powder. Dry Technol..

[61] Goula A.M., Adamopoulos K.G., Kazakis N.A. (2004). Influence of spray drying conditions on tomato powder properties. Dry Technol..

[62] Walton D.E. (2000). The morphology of spray-dried particles a qualitative view. Dry Technol..

[63] Yumoto I., Hirota K., Nodasaka Y., Tokiwa Y., Nakajima K. (2008). Alkalibacterium indicireducens sp nov., an obligate alkaliphile that reduces indigo dye. Int. J. Syst. Evol. Microbiol..

[64] Babaoglu H.C., Bayrak A., Ozdemir N., Ozgun N. (2017). Encapsulation of clove essential oil in hydroxypropyl beta-cyclodextrin for characterization, controlled release, and antioxidant activity. J. Food Process. Preserv..

[65] Rakmai J., Cheirsilp B., Mejuto J.C., Torrado-Agrasar A., Simal-Gandara J. (2017). Physico-chemical characterization and evaluation of bio-efficacies of black pepper essential oil encapsulated in hydroxypropyl-beta-cyclodextrin. Food Hydrocoll..

[66] Corrêa-Filho L.C., Lourenço M.M., Moldão-Martins M., Alves V.D. (2019). Microencapsulation of β-carotene by spray drying: Effect of wall material concentration and drying inlet temperature. Int. J. Food Sci..

[67] de Freitas Santos P.D., Rubio F.T.V., de Carvalho Balieiro J.C., Thomazini M., Favaro-Trindade C.S. (2021). Application of spray drying for production of microparticles containing the carotenoid-rich tucumã oil (*Astrocaryum vulgare* Mart.). LWT.

[68] Garofulic I.E., Kruk V., Martic A., Martic I., Zoric Z., Pedisic S., Dragovic S., Dragovic-Uzelac V. (2020). Evaluation of Polyphenolic Profile and Antioxidant Activity of *Pistacia lentiscus* L. Leaves and Fruit Extract Obtained by Optimized Microwave-Assisted Extraction. Foods.

[69] Ramadan M.F., Amer M.M.A., Sulieman A.E.R.M. (2006). Correlation between physicochemical analysis and radical-scavenging activity of vegetable oil blends as affected by frying of French fries. Eur. J. Lipid Sci. Technol..

[70] Selamat S.N., Mohamad S.N.H., Muhamad I.I., Khairuddin N., Lazim N.A.M. (2018). Characterization of Spray-Dried Palm Oil Vitamin E Concentrate. Arab. J. Sci. Eng..

[71] Sundram K., Sambanthamurthi R., Tan Y.A. (2003). Palm fruit chemistry and nutrition. Asia Pac. J. Clin. Nutr..

[72] Ferreira L.M.D.C., Pereira R.R., de Carvalho F.B., Santos A.S., Ribeiro-Costa R.M., Silva J.O.C. (2021). Green Extraction by Ultrasound, Microencapsulation by Spray Drying and Antioxidant Activity of the Tucuma Coproduct (*Astrocaryum vulgare* Mart.) Almonds. Biomolecules.

[73] Roccia P., Martinez M.L., Llabot J.M., Ribotta P.D. (2014). Influence of spray-drying operating conditions on sunflower oil powder qualities. Powder Technol..

